# Estimating Vaccine-Driven Selection in Seasonal Influenza

**DOI:** 10.3390/v10090509

**Published:** 2018-09-18

**Authors:** Frank T. Wen, Sidney M. Bell, Trevor Bedford, Sarah Cobey

**Affiliations:** 1 Department of Ecology and Evolution, University of Chicago, Chicago, IL 60637, USA; cobey@uchicago.edu; 2 Vaccine and Infectious Disease Division, Fred Hutchinson Cancer Research Center, Seattle, WA 98109, USA; sidneymbell@gmail.com (S.M.B.); trevor@bedford.io (T.B.); 3 Molecular and Cellular Biology Program, University of Washington, Seattle, WA 98195, USA

**Keywords:** strain replacement, indirect effects, universal vaccines

## Abstract

Vaccination could be an evolutionary pressure on seasonal influenza if vaccines reduce the transmission rates of some (“targeted”) strains more than others. In theory, more vaccinated populations should have a lower prevalence of targeted strains compared to less vaccinated populations. We tested for vaccine-induced selection in influenza by comparing strain frequencies between more and less vaccinated human populations. We defined strains in three ways: first as influenza types and subtypes, next as lineages of type B, and finally as clades of influenza A/H3N2. We detected spatial differences partially consistent with vaccine use in the frequencies of subtypes and types and between the lineages of influenza B, suggesting that vaccines do not select strongly among all these phylogenetic groups at regional scales. We did detect a significantly greater frequency of an H3N2 clade with known vaccine escape mutations in more vaccinated countries during the 2014–2015 season, which is consistent with vaccine-driven selection within the H3N2 subtype. Overall, we find more support for vaccine-driven selection when large differences in vaccine effectiveness suggest a strong effect size. Variation in surveillance practices across countries could obscure signals of selection, especially when strain-specific differences in vaccine effectiveness are small. Further examination of the influenza vaccine’s evolutionary effects would benefit from improvements in epidemiological surveillance and reporting.

## 1. Introduction

Vaccination against seasonal influenza is intended to reduce the incidence of disease. Vaccines that protect at least a little against all circulating influenza viruses should reduce prevalence directly by preventing infection in vaccine recipients and indirectly by preventing infection in potential contacts. In randomized controlled trials (RCTs), the trivalent inactivated vaccine directly reduced the risk of clinical infection by 22% (95% CI: 11–41%) in healthy children [1] and 41% (95% CI: 36–47%) in healthy adults [2]. In households and communities, vaccinating children indirectly reduced the risk of influenza infection in unvaccinated individuals by 5–82% [3,4,5,6]. Since annual vaccination coverage in the United States is nearly 76.3% in children aged 6–23 months and 43.3% in adults [7], the effective vaccination coverage (after taking efficacy against clinical infections into account) may be approximately 17% for both age groups. If we assume the vaccine is equally effective against all strains and that protection against clinical infection also protects against transmission, then current vaccination rates in the United States could be expected to reduce prevalence by 38% (Appendix A, Equations (A1)–(A6)). An obvious place to look for an effect of the seasonal vaccine is thus in prevalence, but the prevalence of influenza is not precisely estimated anywhere [1,2]. Because the effectiveness of the influenza vaccine appears to differ between types, subtypes, and clades of influenza, the indirect epidemiological effects of vaccines might be more detectable as changes in the relative abundances of influenza “strains”.

**Figure A1:**
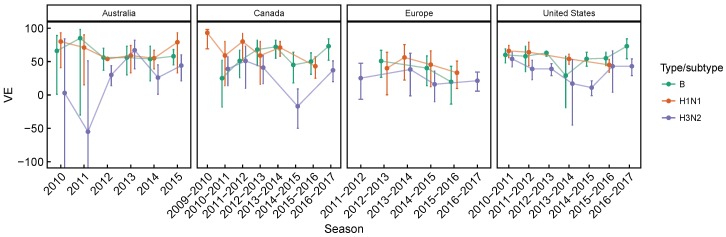
Seasonal vaccine effectiveness by type and subtype measured by test-negative design studies in Australia [24,25,26], Canada [27,28,29,30,31,32,33,34], Europe [35], and the United States [36,37,38,39,40,41,42,43] from the 2009–2010 season to the 2016–2017 season. European VEs include data from study sites in Germany, Spain, France, Croatia, Hungary, Ireland, Italy, the Netherlands, Poland, Portugal, Romania, and Sweden. Error bars show 95% CIs. VE in the United States measures effectiveness against medically attended ARI caused by influenza. Elsewhere, VE measures effectiveness against medically attended ILI caused by influenza. European studies enroll individuals over nine years old, while other studies also enroll individuals from younger age groups.

**Figure A2:**
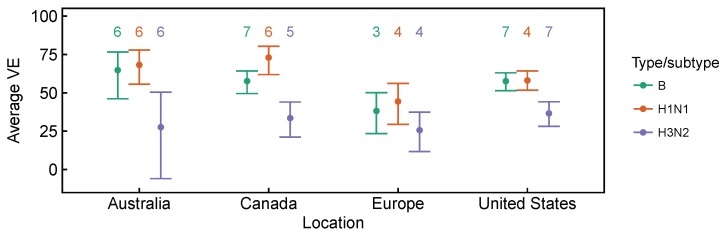
Seasonal VE by type and subtype averaged over time in Australia [24,25,26], Canada [27,28,29,30,31,32,33,34], Europe [35], and the United States [36,37,38,39,40,41,42,43] from the 2009–2010 to the 2016–2017 seasons. European VEs include data from study sites in Germany, Spain, France, Croatia, Hungary, Ireland, Italy, the Netherlands, Poland, Portugal, Romania, and Sweden. Numbers indicate the number of seasons used to calculate each mean. The specific seasons used to compute means are shown in Figure A1. Error bars show 95% CIs. Means and 95% CIs are calculated using arithmetic means of log odds ratios. (Equations (A7)–(A14)).

Differences in vaccine effectiveness (VE) against circulating strains could lead to selection. In theory, vaccines that reduce the transmission of some strains more than others should increase the prevalence of the non-targeted strains relative to the targeted strains [8,9,10,11]. Such vaccine-driven selection has been observed in several pathogens [11,12,13,14,15,16,17], including H5N2 in chickens [18], but it has not yet been reported for seasonal influenza in humans. Studies suggest that seasonal influenza vaccines prevent clinical infection against some strains more than others. RCTs in adults from 2005–2006 to 2008–2009 suggest lower average efficacy over time against H3N2 compared to H1N1 but similar efficacies against H3N2 and B (Table A1 and Table A2) [19,20,21,22,23]. More recent estimates from test-negative design (TND) studies from 2009–2010 to 2016–2017 show lower VE against H3N2 compared to both H1N1 and B on average over time (Figure A1 and Figure A2) [24,25,26,27,28,29,30,31,32,33,34,35,36,37,38,39,40,41,42,43]. For example, TND studies in Canada report average VE of 33.5% (95% CI: 21.2–44.0%) against H3N2, compared to 73.0% (95% CI: 61.9–80.4%) against H1N1 and 57.6% (95% CI: 49.5–64.3%) against B (Appendix B, Equations (A7)–(A14)) [27,28,29,30,31,32,33,34]. In summary, the older RCT studies imply that vaccination should increase the prevalence of H3N2 relative to H1N1, but not necessarily relative to B (Table A3). It is unclear whether these conclusions should apply to recent seasons. More recent evidence from studies based on TND suggest that vaccination should increase the prevalence of H3N2 relative to both H1N1 and B (Table A3). Vaccines might also distinguish between strains defined on other phylogenetic scales, such as lineages of influenza B (Table A4) and clades of influenza A/H3N2 (Table A5) [28,44,45].

**Figure A3:**
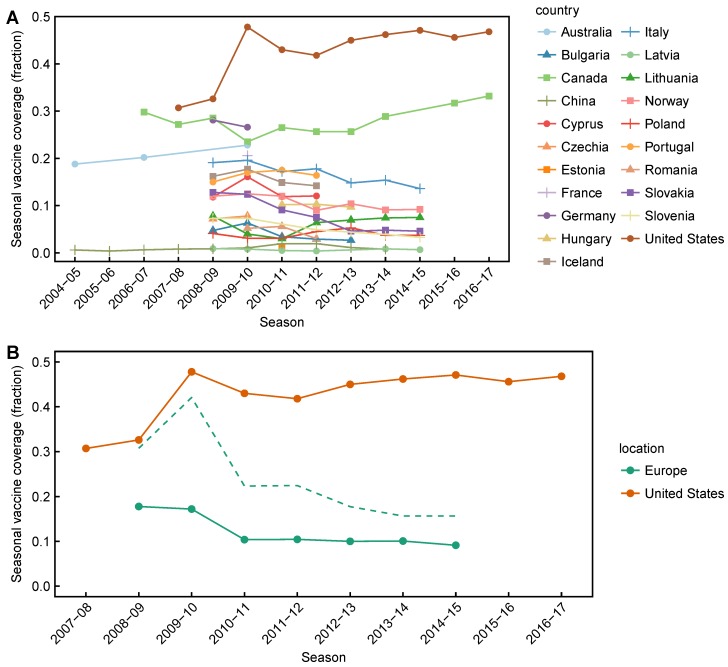
Seasonal vaccine coverage reported by national agencies [7,46,87,88,89]; vaccine coverage in China estimated from doses distributed as reported by the IFPMA IVS task force [90,91]. (**A**) vaccine coverage by country; (**B**) European vaccine coverage compared against United States vaccine coverage. The dashed line shows the total population size of countries reporting vaccine coverage divided by the total population of Europe in each season.

**Figure A4:**
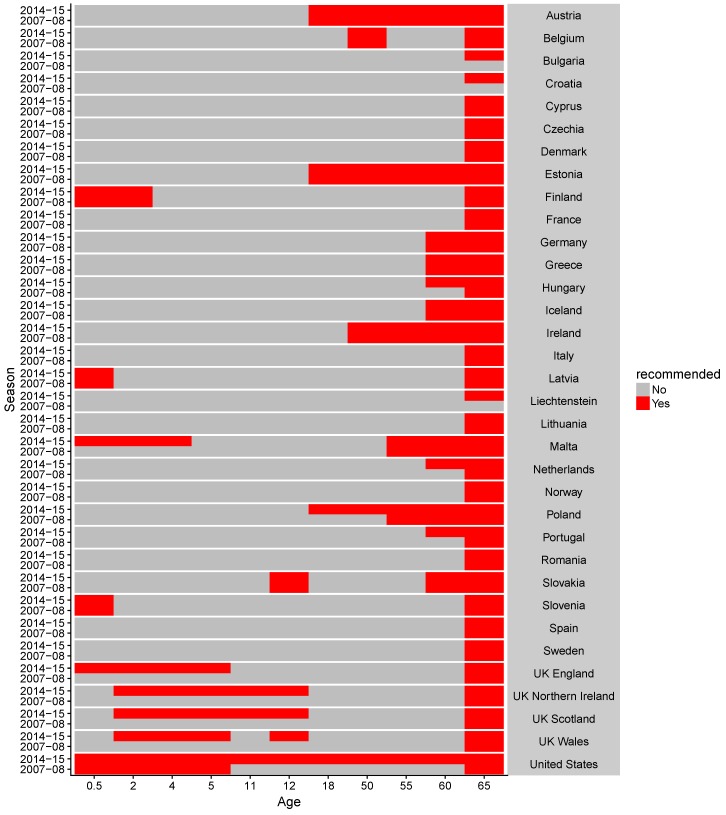
Seasonal influenza vaccination recommendations by age group in Europe [46] and the United States [47] during the 2007–2008 season and the 2014–2015 season.

Higher vaccine coverage should strengthen vaccine-driven selection when VE is different between circulating strains, assuming that vaccination does not eradicate the viral population [8,11] or reduce viral population sizes so much that evolution becomes dominated by genetic drift. Seasonal vaccine coverage has differed consistently between countries over time (Figure A3). For example, in the United States, seasonal vaccine coverage averaged 43.4% and ranged from 32.6% to 46.1% from the 2008–2009 to the 2014–2015 seasons [7]. In contrast, seasonal vaccine coverage in European countries averaged 13.5% (ranging from 10.1% to 18.1% over time) during the same time period [46]. Reported vaccine coverage for any individual European country has not exceeded 30%. Moreover, most European countries do not recommend vaccinating children, in contrast to the United States (Figure A4) [46,47]. Thus, we expect signatures of vaccine-driven selection to be more apparent in the United States compared to Europe. In these temperate populations, annual epidemics are seeded from an external source and go extinct at the end of the season [48,49,50]. Therefore, vaccine-driven selection most likely occurs within individual seasons and on a local scale.

Here, we define expectations for vaccine-driven selection in seasonal influenza based on immunological and epidemiological evidence and test whether these expectations can be detected in available surveillance data. On average, we expect that compared to less vaccinated populations (e.g., European countries [46]), more vaccinated populations (e.g., the United States [7]) will have a lower frequency of the strains that are better targeted by the vaccine. Since seasonality and incomplete mixing lead to regional variation in which strains dominate in each season [51], we compare type and subtype frequencies cumulatively over multiple seasons. We examine selection on three phylogenetic scales: among types or subtypes (H3N2, H1N1, and B), influenza B lineages (B/Victoria and B/Yamagata), and H3N2 clades.

## 2. Materials and Methods

### 2.1. Data Collection

**Figure A5:**
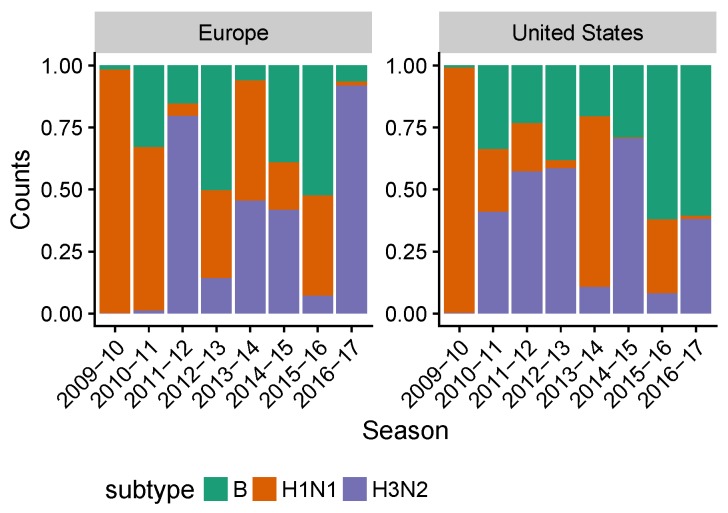
Seasonal subtype frequencies in the United States and Europe. European frequencies are calculated as an average of country-level frequencies, weighted by population size.

**Figure A6:**
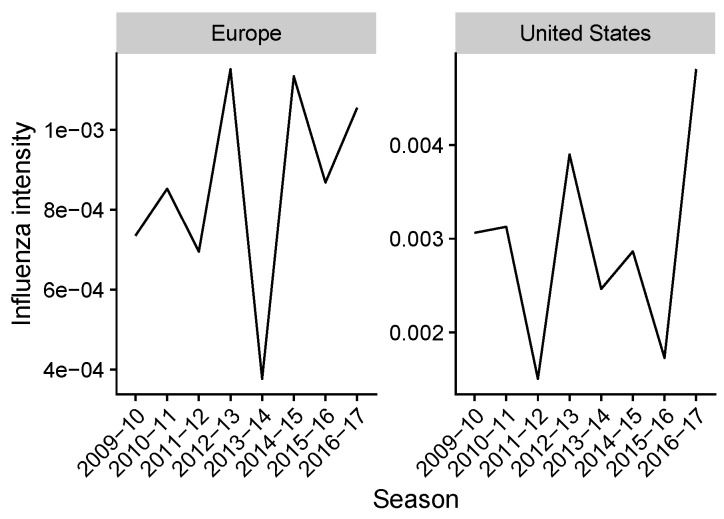
Seasonal influenza intensity in the United States and Europe. European influenza intensity is calculated as a average of country-level influenza intensity, weighted by population size.

We calculated type and subtype frequencies using the numbers of influenza viruses detected by type and subtype, as reported in the in the WHO FluNet [52] database. We also collected influenza-like illness data from the WHO FluID database [53]. We collected surveillance data from the United States, Australia, Canada, China, and all European countries with surveillance data available in the WHO FluNet [52] and FluID databases [53] (Austria, Belgium, Croatia, Denmark, Estonia, Finland, France, Germany, Greece, Hungary, Iceland, Ireland, Italy, Latvia, Lithuania, Norway, Poland, Portugal, Romania, Slovakia, Slovenia, Spain, Sweden, and the United Kingdom). European frequencies are calculated using a population size-weighted sum of country-level frequencies, using census estimates from the United Nations World Population Prospects (Figure A5 and Figure A6). We excluded European countries where sampling was clearly biased towards particular age groups (the Netherlands) or where an influenza-like illness (ILI) denominator was not reported (Malta and Luxembourg) [54]. For the influenza B lineage analysis, we calculated lineage frequencies using the number of sequences identified by lineage as reported in GISAID [55]. For the H3N2 analysis, we collected sequences from GISAID and inferred clade membership and frequencies using Nextstrain [56].

### 2.2. Estimating Influenza Intensity

In calculating cumulative ratios of influenza type, subtype, or lineage incidences (referred to generally as strains hereafter), we first calculate seasonal frequencies of each strain. We then calculate an average frequency over the observation period by taking a sum of seasonal frequencies weighted by influenza intensity. Influenza intensity is derived from ILI incidence and the fraction of laboratory-confirmed influenza-positive respiratory samples.

**Figure A7:**
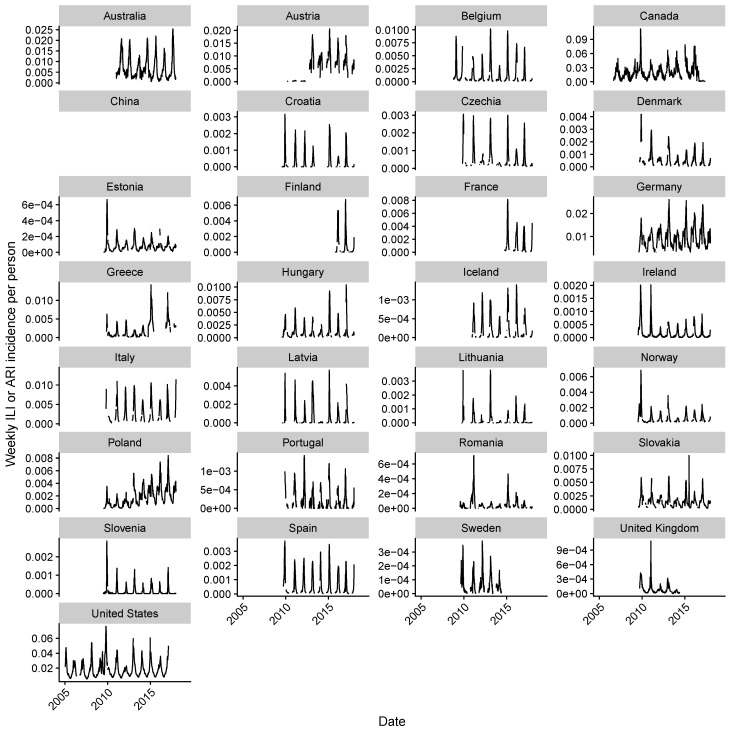
Country-level seasonal influenza-like illness (ILI) or acute respiratory illness (ARI) incidence are shown, as reported in the WHO FluID database [53]. ILI incidence as reported is shown for all countries except for France and Germany. Before the 2014–2015 season, ILI incidence in France is estimated from ARI incidence. ARI incidence is shown for Germany. Incidences are reported at weekly resolution. A fixed *y*-axis scale is shown in Figure A18.

**Figure A8:**
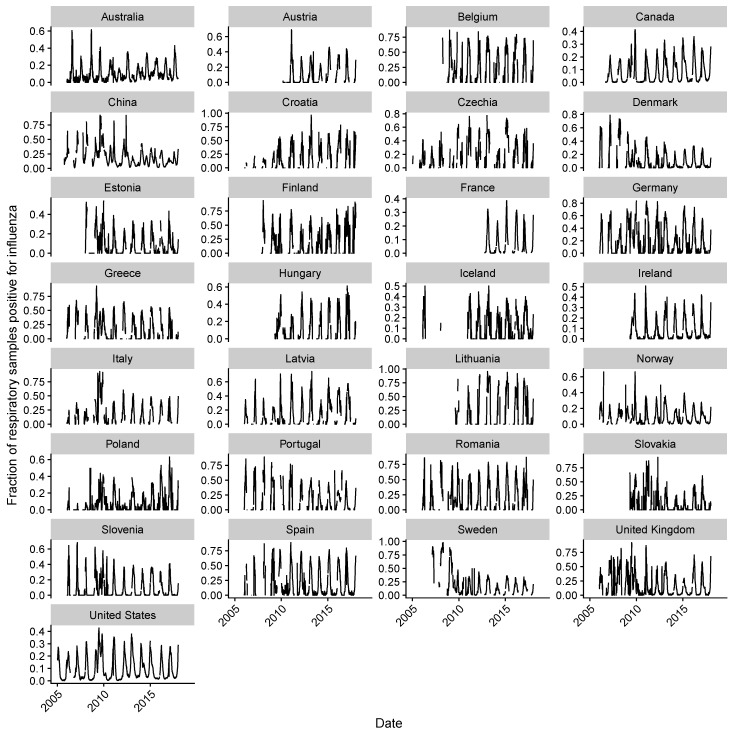
The fraction of laboratory tested influenza positive respiratory samples from National Influenza Centers is shown at weekly resolution, as reported in the WHO FluNet database [52]. A fixed *y*-axis scale is shown in Figure A19.

**Figure A9:**
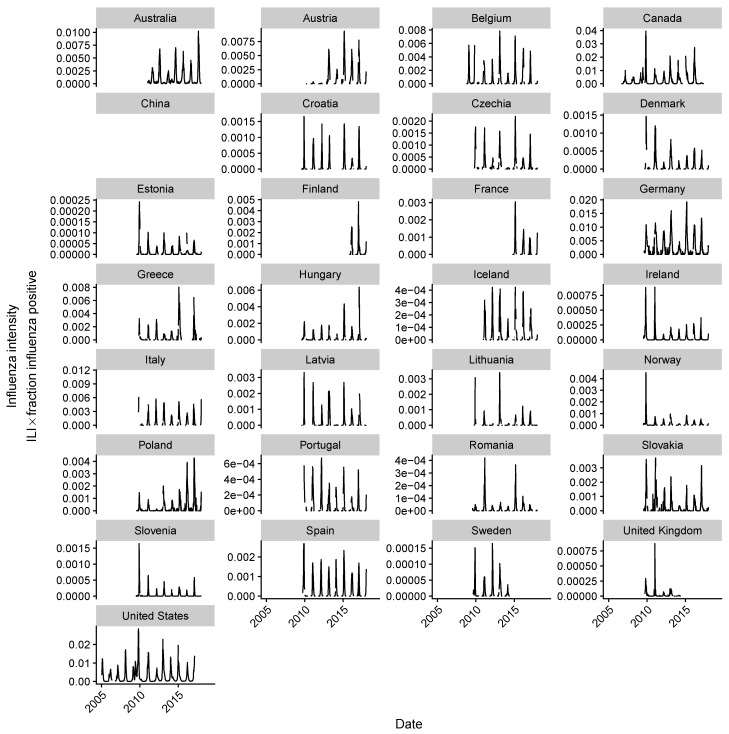
Influenza intensity (ILI × fraction of influenza positive respiratory samples [57]) is shown by country at weekly resolution. For China, the influenza intensity is simply the fraction of influenza positive respiratory samples, since ILI (or any other measure of respiratory illness incidence) is not reported. A fixed *y*-axis scale is shown in Figure A20.

For a given weekly incidence of ILI (Figure A7) and a weekly fraction of laboratory-confirmed influenza-positive respiratory samples (Figure A8), the weekly influenza incidence intensity (Figure A6 and Figure A9) [57], $F_{\mathrm{week}}$, is

(1) $$F_{\mathrm{week}}=\mathrm{ILI}\;\mathrm{incidence}\times \mathrm{fraction}\;\mathrm{of}\;\mathrm{influenza}-\mathrm{positive}\;\mathrm{samples}.$$

The seasonal influenza intensity $F_{x,t}$ is the average weekly influenza intensity over each season for each country *x* and each season *t*. We define a season in the traditional way, starting on week 40 of the year and ending on week 39 of the following year:

(2) $$F_{x,t}\equiv \frac{1}{\mathrm{weeks}}\sum_{\mathrm{weeks}}F_{\mathrm{week}}.$$

**Figure A10:**
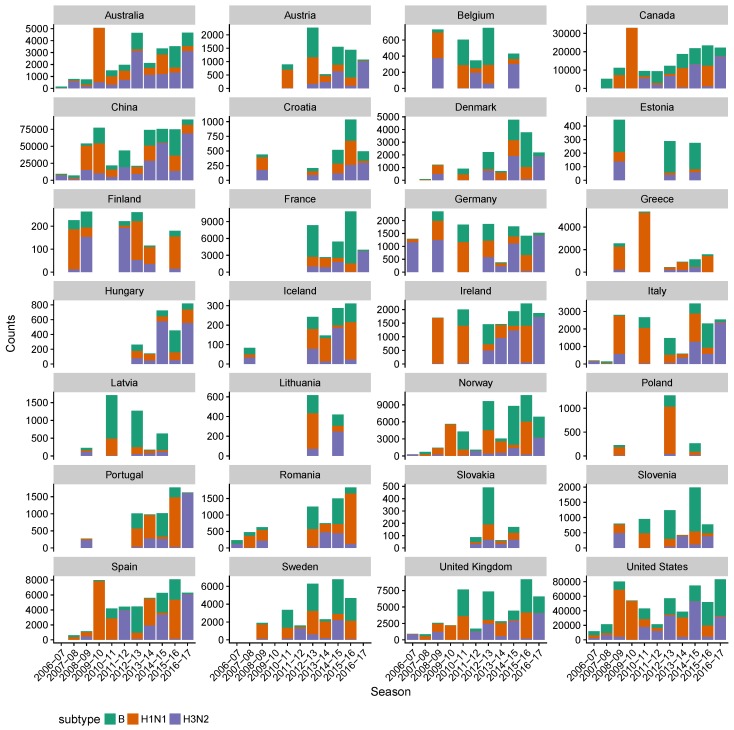
Seasonal counts of laboratory-tested respiratory samples identified by type and subtype, as reported in the WHO FluNet database are shown [52]. These data are used in the type and subtype-level analysis of vaccine-driven selection.

When calculating influenza intensity in Europe, we calculate a sum of European country-level influenza intensities, weighted by population size. The seasonal incidence proxy $I_{x,t,s}$ of strain *s* in season *t* for country *x* is given by the fraction of strain *s* during season *t* (given by $q_{x,t,s}$, Figure A5 and Figure A10) multiplied by the seasonal influenza intensity,

(3) $$I_{x,t,s}=q_{x,t,s}F_{x,t}.$$

Since epidemics are not synchronized across populations [58], we calculate a cumulative incidence ratio for strains $s_{1}$ and $s_{2}$, $I_{s_{1},x}/I_{s_{2},x}$ as the ratio of the influenza intensity-weighted sums of within-season strain frequencies $\sum_{t}^{T}q_{x,t,s}\frac{F_{x,t}}{\sum_{t}F_{x,t}}$ over all seasons *T* where surveillance data are available,

(4) $$\frac{I_{s_{1},x}}{I_{s_{2},x}}=\frac{\sum_{t}^{T}q_{x,t,s_{1}}\frac{F_{x,t}}{\sum_{t}F_{x,t}}}{\sum_{t}^{T}q_{x,t,s_{2}}\frac{F_{x,t}}{\sum_{t}F_{x,t}}}.$$

These equations apply to data from all countries except for China, where ILI data are not reported. We use the fraction of influenza-positive laboratory samples to calculate the influenza intensity for China. For Germany, we use acute respiratory illness (ARI) instead of ILI since ILI is not reported. In France, ARI is reported before the 2014–2015 season and ILI is reported after. We interpolate ILI before the 2014–2015 season by multiplying weekly ARI by the ratio of mean ILI (from 2014–2015 onwards) to mean ARI (from 2009–2010 to 2013–2014).

### 2.3. Power Analysis

**Figure A11:**
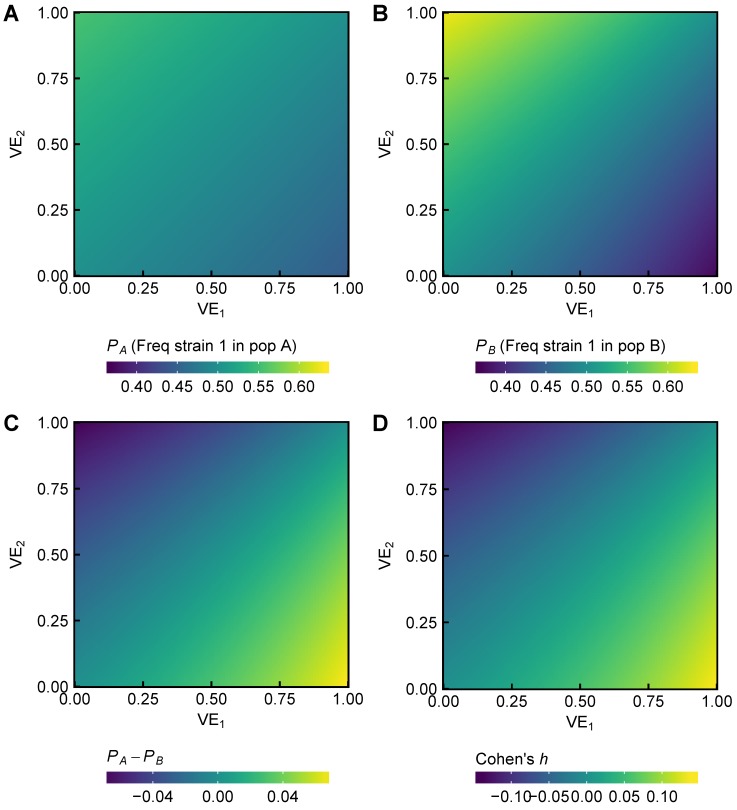
Simulated frequencies of two strains $s_{1}$ and $s_{2}$, circulating in populations *A* (with 20% vaccine coverage) and *B* with (40% vaccine coverage), calculated according to Equation (A23). Given the vaccine effectiveness against both strains, we calculated the fraction of strain 1 (out of strains 1 and 2) in (**A**) population *A* and (**B**) population *B*. The absolute difference in frequencies between regions is shown in (**C**), and the Cohen’s *h* effect size is shown in (**D**). These quantities are used to calculate statistical power in Figure A12.

**Figure A12:**
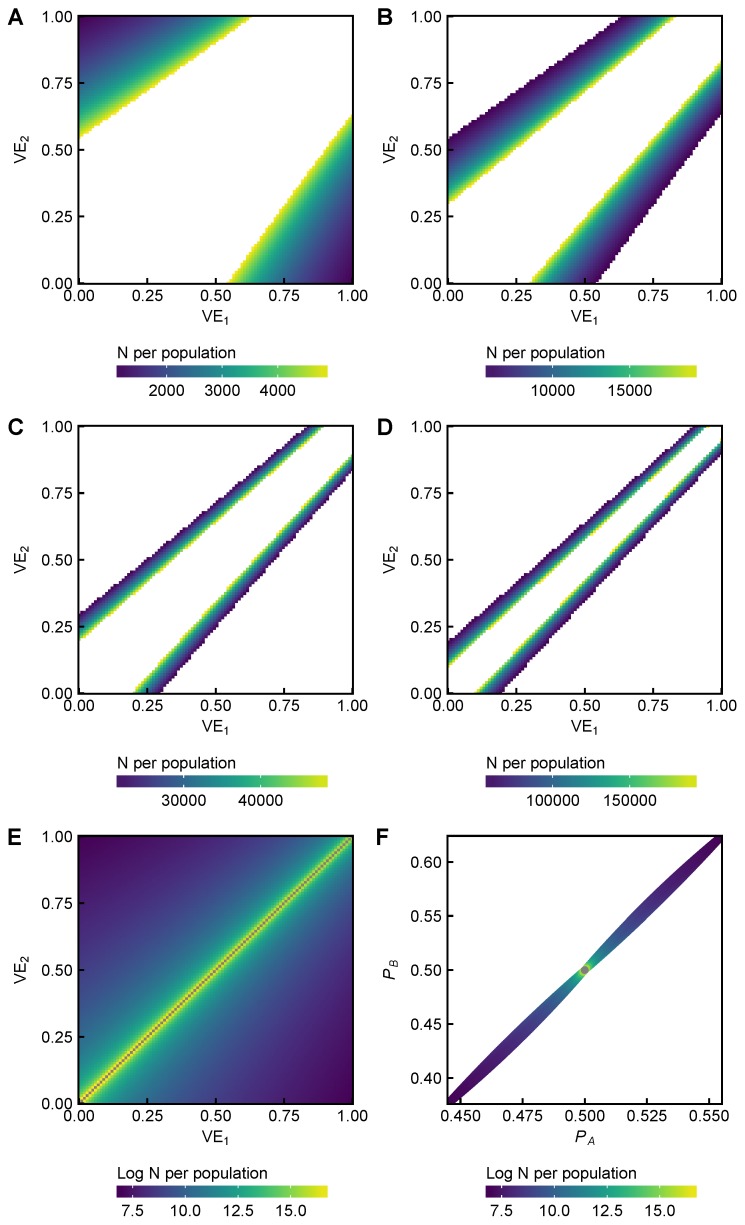
Given differences in vaccine effectiveness against strain 1 and strain 2 (Figure A11), we estimate the sample sizes per population required to achieve 0.90 statistical power to detect the corresponding difference in strain frequencies between populations A (20% vaccine coverage) and B (40% vaccine coverage) at 0.05 significance, assuming equal distribution of sample sizes over time. Required sample sizes are divided into several ranges for visual clarity in (**A**–**D**), and are shown on a log scale in (**E**). The required sample size to detect a difference in the proportion of strain 1 in population A versus population B (which is determined by the difference in VE against strain 1 and 2) is shown on a log scale in (**F**). Also in (**F**), the white space is outside the range of Equation (A23) when VE is between 0 and 1.

We approximate sample sizes required to achieve 0.90 power at 0.05 significance using Pearson’s $\chi^{2}$ test by first assuming that respiratory sample sizes from each season are the same (Figure A11 and Figure A12). We refine these approximations using bootstrapped estimates of power and significance (assuming that pairs of type/subtype abundances are binomially distributed) based on the temporal distributions of sample sizes in the United States. In general, variation in temporal sampling increases the requisite sample size for a given effect size. In the text, we report the sample sizes required, accounting for historical temporal variation in sampling.

### 2.4. Estimating Antigenic Distances between H3N2 Strains and the Vaccine Strain

We inferred the H3N2 phylogenetic tree [56] using a dataset enriched for strains from North America and Europe. We then inferred hemagglutination inhibition (HI) distances to the 2014–2015 vaccine strain (A/Texas/50/2012) for all strains sampled during the 2014–2015 season [56,59]. Epitope distances were calculated as Hamming distances among epitope sites [60].

### 2.5. Data Availability

Data and computer code to replicate the analyses are available at [61].

## 3. Results

### 3.1. Expected Effect Sizes of Vaccination on Selection among Influenza Viruses Vary According to Vaccine Effectiveness

**Figure 1:**
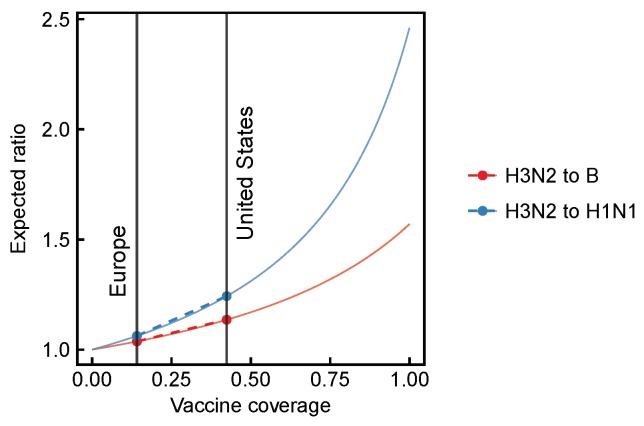
Expected change in the ratios of strains for increasing vaccine coverage. Here, we consider H3N2, H1N1, and B as separate strains. Points show expected subtype ratios at approximate vaccine coverages in the United States and Europe (vertical lines). Dashed lines indicate direct comparisons between expected subtype ratios in the United States and Europe. Here, we assume that subtypes occur at equal frequencies without vaccination, and that VE over multiple seasons is the mean of VEs measured in each season. VE estimates are based on TND studies in Canada [27,28,29,30,31,32,33,34].

We use a simple model to estimate the expected effects of vaccination on the relative abundance of influenza viruses when the vaccine is more effective against some viruses than others (Appendix C, Equation (A15)–(A23)). Given the modest differences in average VE among influenza A/H3N2, A/H1N1, and B [27,28,29,30,31,32,33,34], we expect small to moderate differences in their relative frequencies (Figure 1). The model predicts that relative to H1N1 (73% VE), H3N2 (33.5% VE) should be 1.25 times as abundant in the United States compared to 1.06 times as abundant in Europe (assuming 43% vaccine coverage in the United States and 14% in Europe). Smaller differences in VE, for example between H3N2 (33.5% VE) and B (57.6% VE), generate a smaller expected spatial difference in strain frequencies. Relative to B, we expect H3N2 to be 1.14 times as abundant in the United States compared to 1.04 times as abundant in Europe. Even smaller differences in VE, as we might expect for mismatched influenza B lineages [36,38,39], would cause an even smaller expected spatial difference. Larger differences in VE, for example during the 2014–2015 season between ancestral and mutant H3N2 viruses [28,45], imply a larger difference. This simple model potentially underestimates the vaccines’ effects, since it does not account for indirect effects of herd immunity. In theory, the expected spatial differences could be difficult to detect if small viral population sizes weakened the strength of selection, but there is no evidence that the prevalence of influenza is low or that selection is inefficient [62]. We develop expectations based on our simple model in detail and test for selection using surveillance data at each of the three phylogenetic scales.

### 3.2. Spatial Differences in Influenza Subtype and Type Frequencies Are Not Always Consistent with Vaccine-Driven Selection Caused by Differential Vaccine Effectiveness

We test for vaccine-driven selection among influenza types and subtypes (hereafter referred to generally as subtypes) by comparing the ratios of subtype frequencies from confirmed influenza cases in the United States and Europe from 2009–2010 to 2016–2017. Since vaccine coverage differs consistently between the United States and Europe during these seasons, differences in subtype frequencies between regions would be consistent with vaccine-driven selection. We examine this range of seasons because earlier seasons lack the surveillance data required for the analysis. TND studies in Canada [27,28,29,30,31,32,33,34] and the United States [36,37,38,39,40,41,42,43] over this time period show significantly lower average effectiveness against H3N2 compared to either H1N1 or B (Figure A1 and Figure A2, Equations (A7)–(A14)). VE is also lowest against H3N2 in Europe [35] and Australia [24,25,26], although the local differences in VE by type and subtype are not always statistically significant. From 2008–2009 to 2014–2015, seasonal influenza vaccine coverage in European countries averaged 13.5% [46] compared to 43.4% in the United States [7]. Thus, if vaccines select for subtypes against which the vaccine is less effective, we expect the United States to have a greater proportion of H3N2 relative to H1N1 and relative to B in this period.

We computed influenza subtype frequencies using the number of influenza viruses detected by subtype in the WHO FluNet [52] database. The data are contributed by National Influenza Centers, which test patients’ respiratory samples for influenza positivity, type, and subtype. To account for temporal fluctuations in influenza’s incidence (which is presently not directly measured by surveillance programs), we calculated a weighted average of seasonal subtype and type frequencies (Equation (4)). Frequencies are weighted using an estimated influenza intensity, which is the product of influenza-like illness (ILI) or acute respiratory illness (ARI) incidence and the fraction of influenza-positive respiratory samples (Equation (1), Figure A7, Figure A8 and Figure A9) [57].

**Figure 2:**
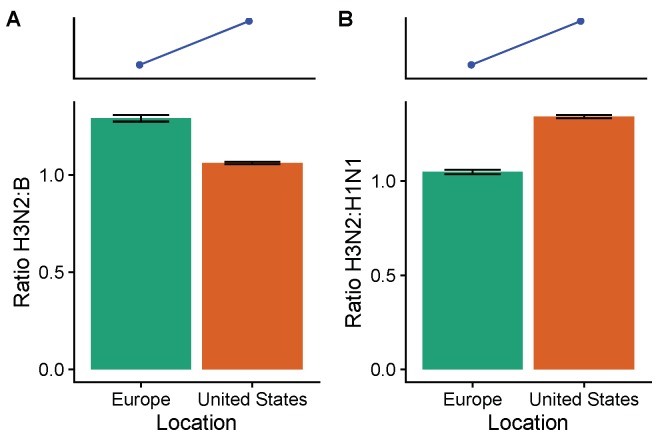
Comparing the ratios of (**A**) H3N2 to B and (**B**) H3N2 to H1N1 between the United States and Europe from the 2009–2010 to the 2016–2017 seasons. Subtype frequencies from the WHO FluNet database are calculated seasonally. The blue lines and points show the expected direction (but not magnitude) of the spatial difference in lineage ratios based on subtype-specific VEs. Ratios are calculated by first averaging seasonal subtype frequencies weighted by the intensity of influenza that season (Equation (4)). Error bars show 95% confidence intervals estimated using multinomial distributions of seasonal subtype frequencies. Unweighted seasonal frequencies are shown in Figure A5, and seasonal influenza intensities are shown in Figure A6.

On average, from the 2009–2010 to the 2016–2017 seasons, H3N2 was less abundant than B and more abundant than H1N1 in the United States compared to Europe (Figure 2). We estimate that H3N2 was 1.06 (95% CI: 1.06–1.07) times more abundant than influenza B in the United States and 1.23 (95% CI: 1.22–1.25) times more abundant than B in Europe. This difference is in the opposite direction expected from TND studies over the study period. Compared to influenza H1N1, H3N2 was 1.34 (95% CI: 1.33–1.35) times as abundant in the United States and 0.97 (95% CI: 0.95–0.98) times as abundant in Europe. This difference is in the expected direction, since vaccines were more effective against H1N1 than H3N2 on average during the study period.

**Figure 3:**
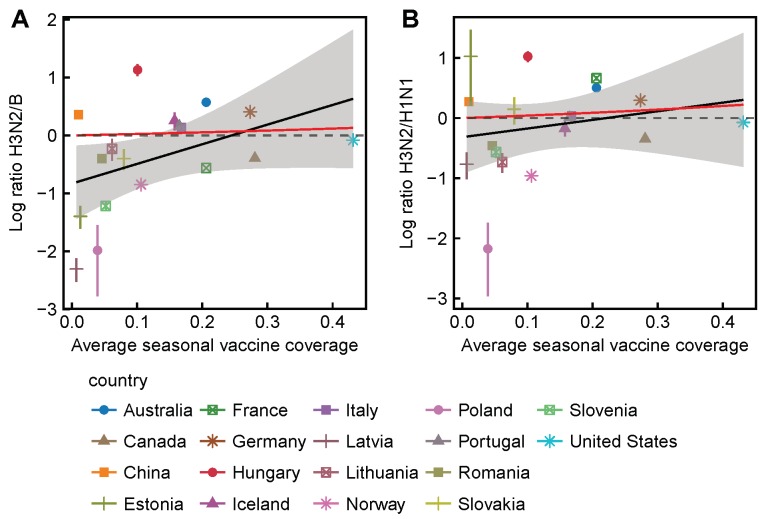
Differences in countries’ subtype ratios are partially consistent with vaccine-driven selection. (**A**) the ratio of H3N2 to B among countries does not significantly correlate with the average seasonal vaccine coverage (Pearson’s r=0.24,p=0.33); (**B**) the ratio of H3N2 to H1N1 among countries significantly correlates with the average seasonal vaccine coverage (Pearson’s r=0.50,p=0.03). Subtype ratios are adjusted for seasonal influenza intensity (Equation (4)). Error bars show 95% confidence intervals estimated using multinomial distributions of seasonal subtype frequencies. Red lines show expectations based on Equation (A23), estimated using VE measured in Canada, and are identical to the trajectories shown in Figure 1. Dashed lines representing no effect of vaccination on subtype ratios are placed for visual reference. The number of seasons contributing to each data point is shown in Figure A14.

**Figure A13:**
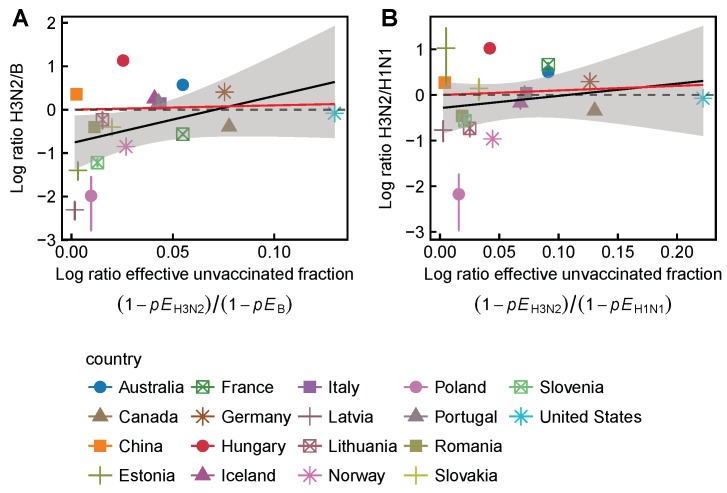
Country-level differences in subtype ratios of countries are not consistent with vaccine-driven selection, accounting for vaccine effectiveness. (**A**) the ratio of H3N2 to B among countries does not significantly correlate with the theoretical ratio (Pearson’s r=0.21,p=0.40); (**B**) similarly, the ratio of H3N2 to H1N1 among countries correlates significantly with the theoretical ratio (Pearson’s r=0.50,p=0.04). Theoretical ratios are calculated using Canadian VEs [27,28,29,30,31,32,33,34] for the Northern Hemisphere and Australian VEs for the Southern Hemisphere [24,25,26]. Error bars show 95% confidence intervals estimated using multinomial distributions of seasonal subtype frequencies. Red line shows expectation based on Equation (A23). The number of seasons contributing to each data point is shown in Figure A14.

We also tested for selection over finer increments of vaccine coverage by testing for a correlation between national vaccine coverage and subtype ratios. Following the same reasoning as before, we expect the ratios of H3N2 to H1N1 to increase monotonically with vaccine coverage. We similarly expect the ratios of H3N2 to B to increase monotonically with vaccine coverage, though to a lesser degree than H3N2 to H1N1. We found a significant correlation between average seasonal vaccine coverage and the ratio of H3N2 to H1N1 (Pearson’s r=0.51,p=0.03) but no significant correlation between coverage and the ratio of H3N2 to B (Pearson’s r=0.24,p=0.34) (Figure 3). Results were similar when adjusting vaccine coverage for VE (Figure A13), using Canadian VE [27,28,29,30,31,32,33,34] for the Northern Hemisphere and Australian VE [24,25,26] for the Southern Hemisphere.

### 3.3. Influenza B Lineage Frequencies Differ Marginally Significantly between More and Less Vaccinated Populations during Seasons Where Only One Lineage Was Included in the Vaccine

Multiple lines of evidence offer conflicting expectations for how the trivalent inactivated vaccine should select for influenza B lineages (Table A4). A quadrivalent vaccine containing viruses from both the B/Yamagata and the B/Victoria lineages was introduced in the 2013–2014 season and currently accounts for ∼80% of all influenza vaccinations in the United States [63]. In clinical trials, the quadrivalent vaccine elicited significantly greater hemagglutination inhibition (HI) titers against both lineages than did the trivalent vaccine against the heterologous lineage [64,65], suggesting that vaccine-induced immunity is partly lineage-specific. Mouse models and studies in children using the live attenuated vaccine suggest that vaccination with a Victoria strain (B/Brisbane/60/2008-like) induces antibody responses against Victoria and Yamagata strains, but vaccination with a Yamagata strain (B/Florida/4/2006-like) only elicits antibody responses against Yamagata [66,67]. Despite these immunological differences measured by HI, the effectiveness of the trivalent vaccine against clinical infection has been comparable against both lineages in the three seasons for which dual estimates exist [36,38,39]. Moreover, trivalent vaccines are effective against influenza B even in seasons dominated by a lineage that mismatches the vaccine [29,34]. Thus, based on TND studies, which measure vaccine-induced protection against clinical influenza infection (albeit with some bias [68]), we expect no difference in the ratios of vaccine-unmatched to matched influenza B lineages between the more vaccinated United States and less vaccinated Europe.

**Figure A15:**
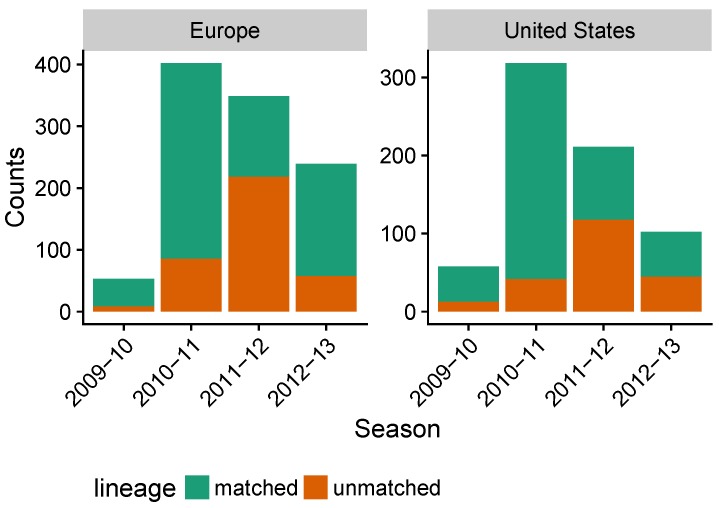
Seasonal counts of influenza B sequences contained in the GISAID database. Colors represent lineages that are matched or unmatched to the vaccine strain in the trivalent inactivated vaccine. These data are used in the influenza B lineage-level analysis of vaccine-driven selection.

We computed influenza B lineage frequencies using sequence data from the GISAID database (Figure A15) [55]. We use sequences instead of virological data from the FluNet database because B lineage typing was not performed on respiratory samples in most countries until after the quadrivalent vaccine was introduced. We examine data from the 2009–2010 to the 2012–2013 seasons (before the introduction of the quadrivalent vaccine), which provide enough sequences to detect a medium-sized difference in B lineage frequencies (Cohen’s h>0.5) with 80% power at 0.05 significance. As in the type- and subtype-level analysis, we attempted to minimize the effects of natural spatiotemporal variation in influenza’s incidence by weighting each season by an estimated influenza intensity.

**Figure 4:**
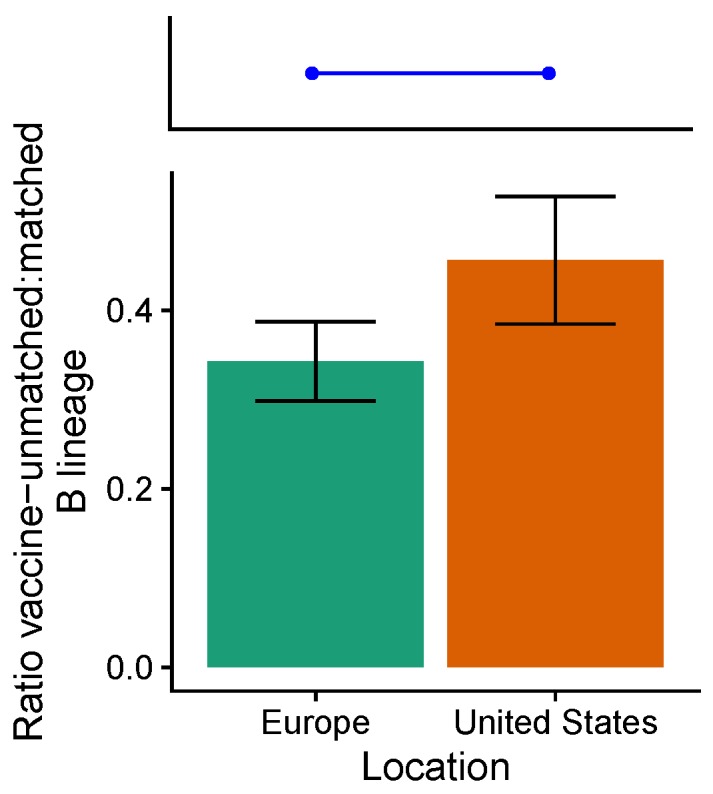
The ratios of vaccine-unmatched to matched B lineages differ marginally between the United States and Europe from the 2009–2010 to the 2012–2013 seasons (p=0.05). The blue line and points show the expectation of no spatial difference in lineage ratios under the assumption that VE does not differ between lineages. Error bars indicate 95% binomial confidence intervals. Unweighted seasonal lineage frequencies are shown in Figure A15.

We found a greater, but marginally nonsignificant (p=0.05), abundance of vaccine-unmatched (non-targeted), relative to vaccine-matched (targeted) influenza B lineages in the United States compared to Europe over this period (Figure 4). We estimated that relative to the vaccine-unmatched lineage, the vaccine-matched lineage was 0.47 (95% CI: 0.39–0.53) times as abundant in the United States and 0.34 (95% CI: 0.30–0.39) times as abundant in Europe. The direction of the effect is consistent with selection for the vaccine-unmatched lineage.

### 3.4. In the 2014–2015 Season, 3c2.A H3N2 Clades Were More Frequent in the United States Than Europe

**Figure 5:**
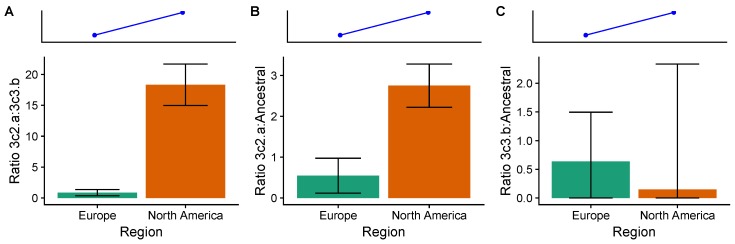
Ratios of (**A**) 3c2.A to 3c3.B, (**B**) 3c2.A to ancestral (3c and 3c3) viruses, and (**C**) 3c3.B to ancestral viruses during the 2014–2015 season in the United States and Europe. The blue lines and points show the expected direction (but not magnitude) of the spatial difference in lineage ratios based on (**A**) clade-specific VEs or (**B**,**C**) antigenic differences between clades. Error bars indicate 95% multinomial confidence intervals. Complete clade frequencies are shown in Figure A16.

We analyzed H3N2 strain frequencies from the 2014–2015 season, where immunological and epidemiological evidence suggests large differences in VE among circulating clades (Table A5). During this season, the trivalent inactivated vaccine contained an A/Texas/50/2012-like H3N2 component, belonging to the ancestral 3c clade. Circulating viruses belonging to the 3c2.A clade had acquired a new glycosylation site and several other amino acid substitutions in the antigenic site B of HA [44]. Viruses in the 3c3.B clade also acquired several amino acid substitutions in antigenic sites [28]. These mutations may have made the vaccine ineffective against 3c2.A strains (VE: −13%; 95% CI, −51% to 15%) and moderately effective against 3c3.B strains (VE: 52%; 95% CI, −17% to 80%) [28,45]. VE against the ancestral clades during 2014–2015 is inestimable due to few cases [45]. Based on clade-specific VE, we expect a greater frequency of 3c2.A viruses relative to 3c3.B in more vaccinated populations compared to less vaccinated populations. We find that, relative to 3c3.B viruses, 3c2.A viruses were 18.3 (95% CI: 15.0–21.7) times as abundant in the United States, compared to 0.86 (95% CI: 0.36–1.35) times as abundant in Europe (Figure 5). This difference is consistent with low VE against 3c2.A compared to moderate VE against 3c3.B.

We also tested for selection based on inferred antigenic phenotypes. Differences in antigenic phenotypes are often estimated using antigenic distances estimated from HI assays using naive ferret antisera [56,59]. Antigenic distances have also been estimated by amino acid Hamming distances among epitope sites [60,69,70]. While estimated antigenic distances are useful for studying general evolutionary patterns, both metrics have unmeasured error that probably varies between seasons and populations. For instance, epitope-based Hamming distances could underestimate the immunological effects of glycosylation sites, which easily disrupt antibody binding [44]. For HI distances, antisera raised in naive ferrets can have different specificities compared to antisera from humans, because previous exposures affect the generation of new immune responses [71,72,73]. Nonetheless, traditional measures of antigenic distance partly correlate with VE [69,70]. Strains from the 2014–2015 season carrying mutations in the antigenic site B of HA reduced the binding of antibodies elicited by vaccination with A/Texas/50/2012 in both ferrets and humans [44], suggesting agreement between ferret HI titers and VE in humans.

Although VE against ancestral H3N2 clades during the 2014–2015 season is inestimable, antigenic distances suggest that vaccination should select for both 3c2.A and 3c3.B relative to ancestral H3N2 viruses. Both 3c2.A and 3c3.B viruses are antigenically distant from the ancestral 3c and 3c3 viruses (which contained the H3N2 vaccine component) [28,44,56], suggesting that vaccine-induced immunity might protect less against the mutant viruses [69,70]. Consistent with vaccine-driven selection, relative to ancestral 3c and 3c3 viruses, 3c2.A viruses were 2.75 (95% CI: 2.22–3.28) times as abundant in the United States, compared to 0.54 (95% CI: 0.12–0.97) times as abundant in Europe (Figure 5). However, relative to ancestral 3c and 3c3 viruses, 3c3.B viruses were 0.15 times as abundant (95% CI: 0.00–2.35) in the United States compared to 0.64 times as abundant (95% CI: 0.00–1.50) as abundant in Europe (Figure 5). The nonsignificant difference is in the opposite direction of expectations based on antigenic differences, although there is large uncertainty in the ratios due to low abundance of ancestral viruses. Together, these results show support for vaccine-driven selection for 3c2.A relative to ancestral viruses, but not 3c3.B relative to ancestral viruses.

**Figure A17:**
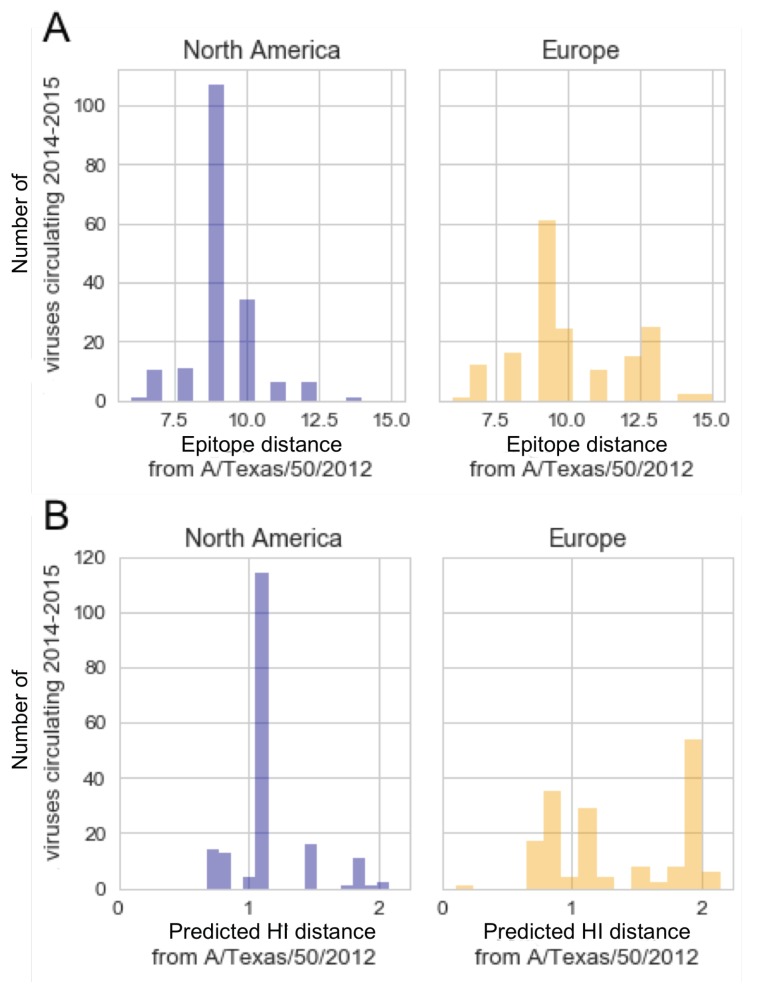
Distributions of circulating H3N2 strains with given antigenic distances from the 2014–2015 vaccine strain (A/Texas/50/2012), stratified by region. Antigenic distances in (**A**) are calculated as Hamming distances between epitope sites, as defined in [60]. H3N2 strains in North America were more antigenically distant from the vaccine strain by epitope Hamming distance (9.2 units, 95% CI: 9.0–9.4) compared to Europe (10.0 units, 95% CI: 9.7–10.3). Antigenic distances in (**B**) are calculated using HI titers [56,59]. North American H3N2 strains are significantly less distant from the vaccine strain (1.17 units, 95% CI: 1.12–1.21) compared to Europe (1.34 units, 95% CI: 1.26–1.42).

To test for vaccine-induced selection at a the level of individual genotypes, we estimated the antigenic distances between the vaccine strain and H3N2 strains circulating in the 2014–2015 season. As before, if vaccination selected for mutant H3N2 strains during the 2014–2015 season, then we would expect circulating strains in more vaccinated populations to be more antigenically distant from the vaccine strain compared to less vaccinated populations. We find that by two measures of antigenic distance, frequencies of circulating H3N2 strains and the vaccine strain (A/Texas/50/2012) in North America and Europe are not consistent with vaccine-driven selection in the 2014–2015 season (Figure A17). During the 2014–2015 season, H3N2 strains in North America were antigenically less distant from the vaccine strain by epitope Hamming distance (9.2 units, 95% CI: 9.0–9.4) compared to Europe (10.0 units, 95% CI: 9.7–10.3), opposite of expectations. Similarly, according to ferret-derived HI distance, North American H3N2 strains were significantly less distant from the vaccine strain (1.17 units, 95% CI: 1.12–1.21) compared to Europe (1.34 units, 95% CI: 1.26–1.42), also opposite of expectations. Thus, although clade frequencies are consistent with vaccine-driven selection among H3N2 strains in the 2014–2015 seasons, conventional measures of antigenic distances between circulating H3N2 strains are not consistent with expectations.

### 3.5. Power Analysis

Is the inconsistent support for vaccine-induced evolution evidence of the vaccine’s weak effects or a consequence of insufficient data? We conducted a power analysis using VE from TND studies conducted in Canada during the time period that we analyzed (2009–2010 to 2016–2017) [27,28,29,30,31,32,33,34]. We first computed the expected difference in subtype and type proportions between two populations (Equation (A23)), one vaccinated at 20% and the other at 40% (representing the Europe and the United States, respectively). We assume VEs of 34% against H3N2, 58% against B, and 73% against H1N1. The expected proportion of H3N2 out of H3N2 and B is 51.3% in Europe versus 53.0% in the United States (or H3N2:B ratios of 1.05 and 1.12). The expected proportion of H3N2 out of H3N2 and H1N1 is 52.2% in Europe versus 55.0% in the United States (or H3N2:H1N1 ratios of 1.09 and 1.22). For any given sample of influenza viruses from two populations, one vaccinated at 20% and one at 40% (representing the Europe and the United States, respectively), ∼10,000 samples per population are needed to detect the expected spatial difference in the relative abundance of H3N2 to H1N1, whereas ∼28,000 samples per population are needed to detect the expected difference for H3N2 to B at 0.90 power and 0.05 significance (Pearson’s $\chi^{2}$ test, Figure A11 and Figure A12). For a difference in VE comparable to those among H3N2 clades in 2014–2015 (about 50% against 3c2.A and 0% 3c3.B), ∼6000 samples per population would be necessary to detect a difference in frequencies at 0.90 power and 0.05 significance. However, although large sample sizes are needed to detect weak signals of vaccine-driven selection, different surveillance practices among countries [54,74] potentially bias strain frequencies in ways that obscure vaccination’s effects and are difficult to measure.

The present sample sizes from 2006–2007 to 2016–2017 are more than large enough to detect expected differences in the relative abundances of H3N2 to H1N1 and B between the United States and Europe. However, for the B lineage analysis, the number of sequences available from 2009–2010 to 2012–2013 is insufficient to detect even the maximum expected difference in proportions (i.e., 100% effectiveness against one lineage and 0% against the other, implying vaccine-unmatched lineage prevalences of 0.56 in the less vaccinated population and 0.63 in the more vaccinated population) at 0.90 power and 0.05 significance. Given the number of available sequences in the United States and Europe, the power to detect the maximum difference in B lineage proportions is ∼0.60 at 0.05 significance. For the H3N2 analysis, the power to detect the expected difference in clade frequencies (assuming 50% effectiveness against one clade and 0% against the other) at 0.05 significance is ∼0.74, although the actual difference in H3N2 clade proportions exceeds what is predicted by our model (Equation (A23)). Statistical power may be larger in future seasons, assuming surveillance continues. For example, ∼6000 H3N2 sequences are available in GISAID from the 2016–2017 season, which would have been sufficient to detect the expected difference in H3N2 clade proportions based on VEs from the 2014–2015 season (at 0.90 power and 0.05 significance).

## 4. Discussion

We detected partial evidence of vaccine-driven selection on seasonal influenza. At the type and subtype level, TND studies from the 2009–2010 to the 2016–2017 seasons suggest that the vaccine has been less effective against H3N2 than against B or H1N1. Thus, we expect more vaccinated populations to have a greater proportion of H3N2 compared to less vaccinated populations during these seasons. Contrary to expectations, we find that H3N2 is relatively less common than B in the more vaccinated United States compared to Europe during this time period. However, consistent with expectations, we find that H3N2 is significantly more frequent relative to H1N1 in the United States compared to Europe during this period, and there was also a consistent trend of higher H3N2 to H1N1 ratios in more vaccinated countries. When we examined influenza B, we found marginally significant differences in the ratios of vaccine-matched and unmatched lineages between the United States and Europe, though small sample sizes limit statistical power. It is unclear if we should expect differences given the apparently high cross-protection after vaccination. Lastly, during the 2014–2015 influenza season, the vaccine was ineffective against the H3N2 3c2.A clade, which carried several antigenic mutations, but moderately effective against the 3c3.B clade. We found that strains belonging to the 3c2.A clade were significantly more frequent in North America compared to Europe, suggesting vaccine-driven selection during this season. However, alternative measures of antigenic distance between strains in these regions were not consistent with vaccine-driven selection. Collectively, these results indicate that vaccine-driven selection could be influencing the frequencies of influenza A subtypes, and the distribution of H3N2 clades in one season is also consistent with vaccine-driven selection. However, evidence of vaccine-induced selection on or within influenza B is less clear. In general, we find better support for expectations involving selective effects that are moderate to large, based on VE (i.e., between H3N2 and H1N1 and among H3N2 clades) rather than small (i.e., between H3N2 and B and between B lineages).

The analysis suggests that VE measured from 2009–2010 to 2014–2015 does not explain the relative frequencies of influenza A/H3N2 and B viruses over the same time period. Although VEs measured by TND during the period of study (2009–2010 to 2016–2017) are lower to H3N2 than B [24,25,26,27,28,29,30,31,34,35,36,37,38,39,40,41,42,43], estimates based on TND studies and RCTs from earlier seasons (2005–2006 to 2008–2009) show comparable effectiveness [19,20,21,22,23,32,33] (Appendix B). If earlier studies suggesting comparable VE against H3N2 and B are relevant to recent seasons, then H3N2 might be expected to be comparably frequent relative to B, instead of more frequent. Smaller differences in VE would also lead to effect sizes that are small in comparison to the variation observed in surveillance data.

The higher H3N2 to H1N1 ratio in more vaccinated populations compared to less vaccinated populations suggests a greater difference in VE between H3N2 and H1N1 compared to H3N2 and B. Unlike VE measurements for influenza B, VE measurements for H1N1 (RCTs from early seasons and TND studies from early and recent seasons) are consistently higher than for H3N2 (Appendix B) [19,20,21,22,23,24,25,26,27,28,29,30,31,32,33,34,35,36,37,38,39,40,41,42,43], suggesting that differences in VE between influenza A subtypes persist through time. Spatial differences in subtype frequencies might only be detectable when differences in subtype-specific VE are consistently large.

Unmeasured bias in strain frequency data adds uncertainty to our analysis. In general, uncertainty in subtype and type frequencies at a regional scale is small due to large sample sizes. However, strains associated with more severe disease (e.g., H3N2 [75]) may be reported more frequently, since testing for subtype and type draws from symptomatic and medically attended influenza cases. Accordingly, H3N2 may be overrepresented in countries that have larger at-risk demographic groups compared to countries that have smaller at-risk groups. H3N2 may also be overrepresented in countries that use ARI or severe ARI case definitions to screen for influenza (e.g., France before 2014–2015 and Germany [54]), since these cases are more severe than ILI. Thus, compared to what we measured, the ratio of H3N2 to B in Europe may be more similar to that in the United States, and the ratio of H3N2 to H1N1 in Europe may be even lower than that in the United States. Differences in surveillance practices between countries might also contribute to the large variation in subtype frequencies (relative to expectations) that we observe at a national scale, thus obscuring signals of vaccine-driven selection.

Although our analysis attributes all error to variation in strain frequencies, there is also error and potentially bias in VE measurements. Conventional VE measures effectiveness against clinical influenza infection and may fail to capture effectiveness against typical influenza infections due to case ascertainment bias [68]. True VE is thus potentially lower than reported against viruses causing less severe disease, which would make VE between H3N2, B, and H1N1 more comparable. If the VEs are more similar, then we would expect that the type and subtype ratios would be less affected by vaccine coverage, which is partly consistent with what we observe for H3N2 to B. If vaccines are less effective at preventing infections, then they may also be less effective at preventing transmission. Prospective randomized case-control studies that estimate the rate of paucisymptomatic and asymptomatic infections and shedding could improve the accuracy of VE measurements.

Future analyses of vaccine-driven selection would benefit from improvements in conceptualization and two areas of influenza surveillance: accurate measurement of VE and standardized surveillance among study populations. Our analysis assumes the largest effects of vaccination are direct: H1N1 cases should be diminished because the vaccine directly protects against severe H1N1 infection. In practice, this reduction in cases could lead to reduced transmission, which should amplify the expected effects on prevalence. However, these effects could be modulated in complicated ways if influenza subtypes and types compete asymmetrically through natural infection or vaccination. Herd immunity, asymmetric cross-immunity, and differences in $R_{0}$ could modify expectations [76]. The nature and strength of these epidemiological variables remain important areas for research. With respect to surveillance, as mentioned, RCTs with frequent testing for influenza infection would help accurately measure effectiveness in preventing infection. Direct comparisons between VE measured by RCTs and TND studies in the same population could inform the reliability of TND-based estimates [77]. VE studies should also include sufficiently large sample sizes to measure age- and type/subtype-specific VE. Standardized surveillance protocols would minimize systematic biases in strain frequencies. Well-documented surveillance protocols (e.g., [74]) and annotated metadata, including patient age and vaccination history, would also help models adjust for differences in subject populations. For these reasons, another place to test for vaccine-driven selection may be between regions of the United States, where surveillance is more consistent and mixing is not rapid enough to homogenize strain compositions [58]. In summary, improvements to our understanding of strain competition and VE measurements will better inform expectations for how vaccines should affect selection, and standardization of surveillance practices would remove a major source of unmeasured bias in surveillance data.

## Figures and Tables

**Figure A14:**
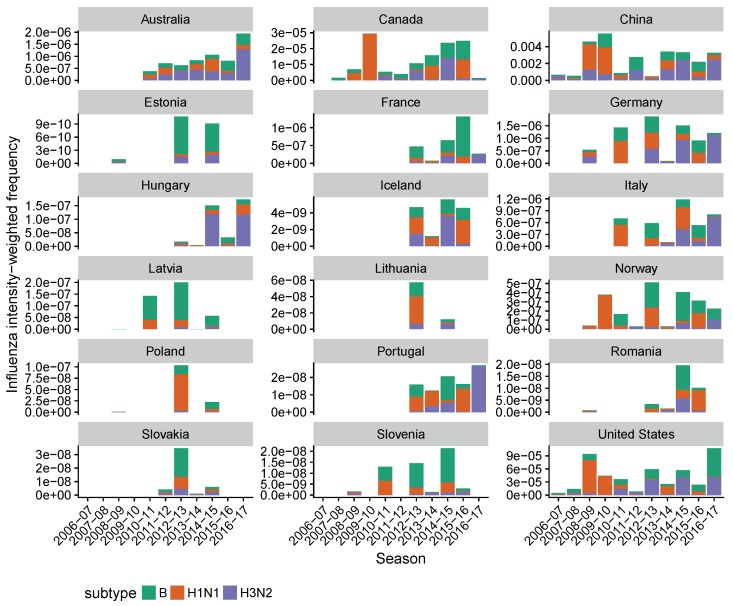
Influenza intensity-weighted seasonal subtype and type frequencies prior to summation for use in country-level analysis (Figure A13 and Figure 3).

**Figure A16:**
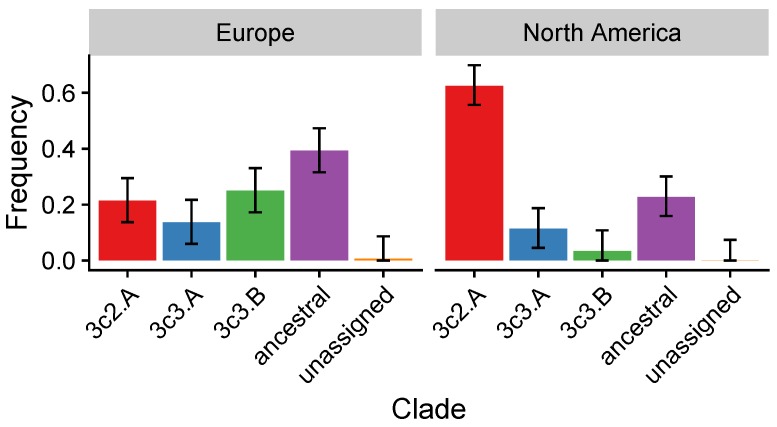
Frequencies of H3N2 clades circulating during the 2014–2015 season, stratified by region. Error bars indicate 95% multinomial confidence intervals. Notably, 3c2.A strains are significantly more frequent in North America compared to Europe, and 3c3.B strains are significantly less frequent in North America compared to Europe.Frequencies of H3N2 clades circulating in the 2014-2015 season, stratified by region

**Figure A18:**
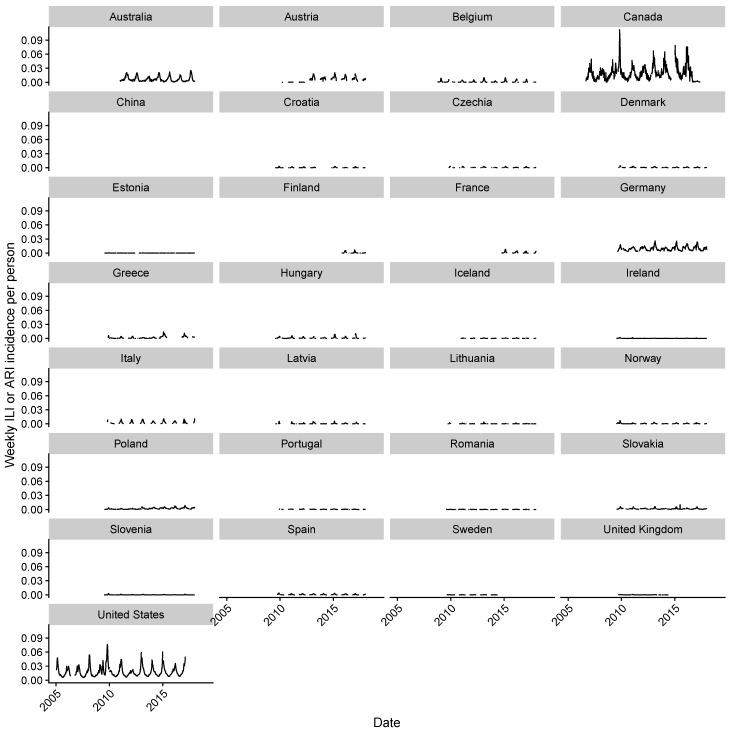
As in Figure A7, but using a fixed *y*-axis scale for visualization.

**Figure A19:**
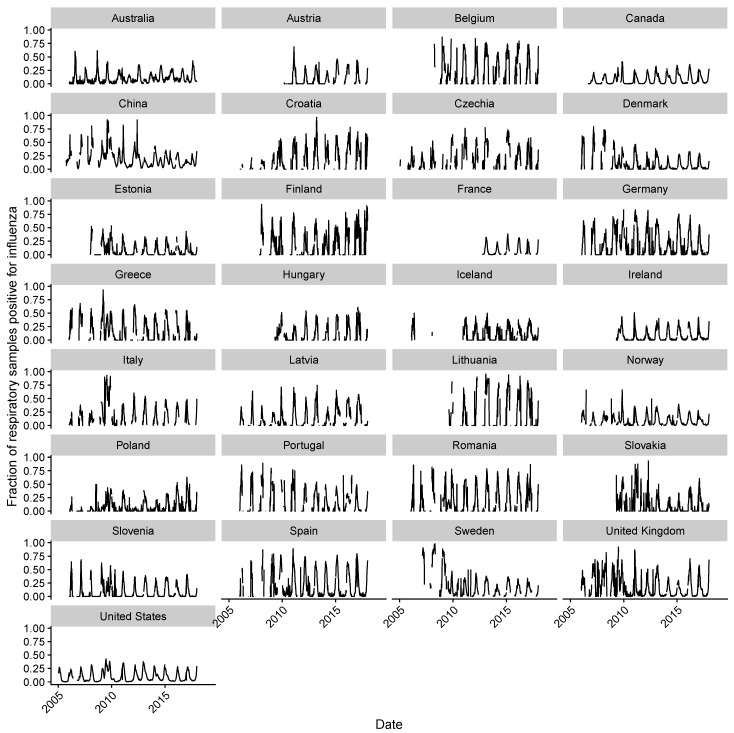
As in Figure A8, but using a fixed *y*-axis scale for visualization.

**Figure A20:**
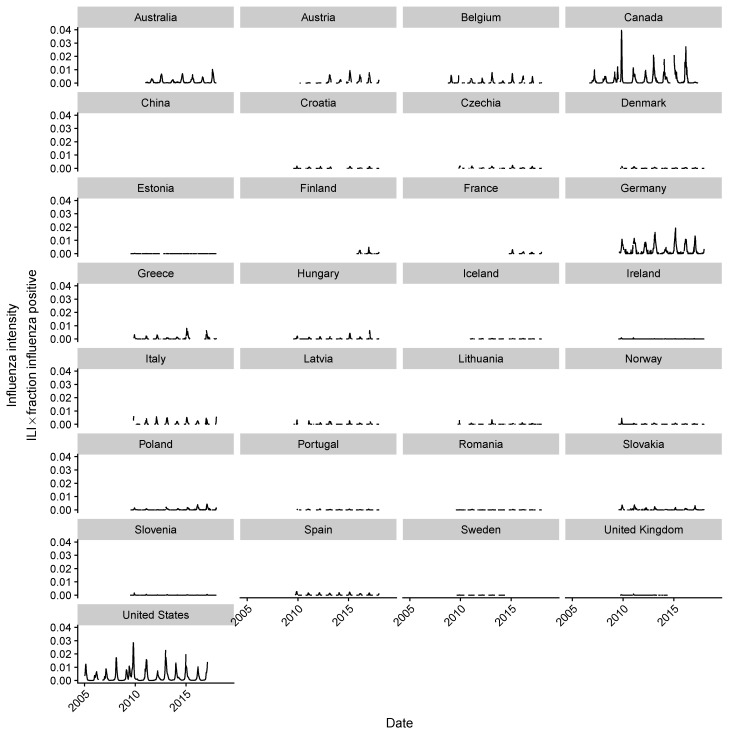
As in Figure A9, but using a fixed *y*-axis scale for visualization.

## Appendix A. Approximate Effects of Vaccination on Prevalence

We derive the approximate impact of vaccination on prevalence using a susceptible, infected, recovered (SIR) compartmental model. *S*, *I*, and *R* represent the fraction of susceptible, infected, and recovered individuals, such that S+I+R=1. The birth rate μ and the death rate are equal, so the population size is constant. All individuals are born into the susceptible class. Transmission and contact occurs at rate β, and recovery occurs at rate γ. We vaccinate some fraction *p* of newborns. Vaccinated individuals move into the recovered class:

(A1) $$\begin{array}{cc} \frac{dS}{dt} & =\mu (1-p)-\beta SI-\mu S, \end{array}$$

(A2) $$\begin{array}{cc} \frac{dI}{dt} & =\beta SI-\gamma I-\mu I, \end{array}$$

(A3) $$\begin{array}{cc} \frac{dR}{dt} & =\gamma I-\mu R+\mu p. \end{array}$$

The endemic equilibrium of $S_{\mathrm{eq}}$, $I_{\mathrm{eq}}$, and $R_{\mathrm{eq}}$ is

(A4) $$\begin{array}{cc} S_{\mathrm{eq}} & =\frac{\gamma +\mu }{\beta }\equiv \frac{1}{R_{0}}, \end{array}$$

(A5) $$\begin{array}{cc} I_{\mathrm{eq}} & =\frac{\mu }{\beta }\left(R_{0}\left(1-p\right)-1\right), \end{array}$$

(A6) $$\begin{array}{cc} R_{\mathrm{eq}} & =1-\frac{1}{R_{0}}-\frac{\mu }{\beta }\left(R_{0}\left(1-p\right)-1\right), \end{array}$$

where $R_{0}\equiv \frac{\beta }{\gamma +\mu }$.

We assume the following parameters for influenza: β=0.36 days${}^{-1}$, γ=0.2 days${}^{-1}$ (i.e., a 5-day duration of infection), and μ=1/30 years${}^{-1}$ (implying $R_{0}=1.8$). The prevalence without vaccination is $2.03\times 10^{-4}$. At 17% vaccine coverage, the prevalence is $1.25\times 10^{-4}$, equivalent to a 38% reduction in prevalence.

## Appendix B. Estimating Average Vaccine Effectiveness by Subtype

Vaccine effectiveness (*E*) from TND studies is expressed as 1−oddsratio:

(A7) $$\begin{array}{c} E=1-\frac{P(\mathrm{infected}\;|\;\mathrm{vaccinated})/P(\mathrm{not}\;\mathrm{infected}\;|\;\mathrm{vaccinated})}{P(\mathrm{infected}\;|\;\mathrm{not}\;\mathrm{vaccinated})/P(\mathrm{not}\;\mathrm{infected}\;|\;\mathrm{not}\;\mathrm{vaccinated})}. \end{array}$$

Vaccine efficacy (*F*) from RCTs is expressed as 1−riskratio:

(A8) $$\begin{array}{c} F=1-\frac{P(\mathrm{infected}\;|\;\mathrm{vaccinated})}{P(\mathrm{infected}\;|\;\mathrm{not}\;\mathrm{vaccinated})}. \end{array}$$

In this section, we refer to both metrics as vaccine effectiveness (*E*) for simplicity.

To calculate average vaccine effectiveness, we assume that the log odds ratios, log(1−E), are approximately normally distributed. Then, the mean effectiveness over *T* seasons *t* is

(A9) $$\begin{array}{cc} \log(1-\bar{E}) & =\frac{1}{T}\sum_{t}^{T}\log\left(1-E_{t}\right), \end{array}$$

(A10) $$\begin{array}{cc} \bar{E} & =1-\exp[\frac{1}{T}\sum_{t}^{T}\log\left(1-E_{t}\right)]. \end{array}$$

Given 95% confidence intervals for seasonal effectivenesses $\left(E_{t,l},E_{t,u}\right)$, we calculate a 95% confidence interval for the average effectiveness $\left({\bar{E}}_{t,l},{\bar{E}}_{t,u}\right)$ by first calculating the 95% confidence interval for the average log odds ratio $\left(\log\left(1-{\bar{E}}_{t,l}\right),\log\left(1-{\bar{E}}_{t,u}\right)\right)$. Again, we assume a normal approximation. For the lower bound,

(A11) $$\begin{array}{cc} \log(1-{\bar{E}}_{l}) & =\log\left(1-\bar{E}\right)+\frac{1}{T}{\left[\sum_{t}^{T}{\left(\log\left(1-E_{t,l}\right)-\log\left(1-E_{t}\right)\right)}^{2}\right]}^{\frac{1}{2}}, \end{array}$$

(A12) $$\begin{array}{cc} \bar{E_{l}} & =1-\exp\left(\log\left(1-\bar{E}\right)+\frac{1}{T}{\left[\sum_{t}^{T}{\left(\log\left(1-E_{t,l}\right)-\log\left(1-E_{t}\right)\right)}^{2}\right]}^{\frac{1}{2}}\right). \end{array}$$

Similarly, for the upper bound,

(A13) $$\begin{array}{cc} \log(1-{\bar{E}}_{u}) & =\log\left(1-\bar{E}\right)-\frac{1}{T}{\left[\sum_{t}^{T}{\left(\log\left(1-E_{t,u}\right)-\log\left(1-E_{t}\right)\right)}^{2}\right]}^{\frac{1}{2}}, \end{array}$$

(A14) $$\begin{array}{cc} \bar{E_{u}} & =1-\exp\left(\log\left(1-\bar{E}\right)-\frac{1}{T}{\left[\sum_{t}^{T}{\left(\log\left(1-E_{t,u}\right)-\log\left(1-E_{t}\right)\right)}^{2}\right]}^{\frac{1}{2}}\right). \end{array}$$

In Australia [24,25,26], Canada [27,28,29,30,31,32,33,34], Europe [35], and the United States [36,37,38,39,40,41,42,43], TND studies from 2009–2010 to 2016–2017 consistently show lower VE averaged over time against H3N2 compared to H1N1 or B (Figure A1 and Figure A2). In Canada and the United States, these differences are statistically significant. In Australia, VE is nonsignificantly lower against H3N2 than B. In Europe, the differences by subtype are consistent with the general patterns, but are not statistically significant. Fewer seasons of VE data in Europe (3–4 seasons on average compared to 4–7 in other locations) reduce the power to detect significant differences in VE by subtype there.

VE measurement protocols differ by location. VE measures effectiveness against ARI caused by influenza in the United States, but measures effectiveness against ILI caused by influenza elsewhere. Additionally, TND studies in Europe measure VE in people over nine years old, while TND studies elsewhere include people from younger age groups. For our VE-adjusted analysis (Figure A13), we use Canadian VEs for Northern hemisphere countries and Australian VEs for Southern hemisphere countries, since studies from these countries offer the best combination of the number of seasons available and consistency in study protocol.

RCTs are only available from earlier seasons (2005–2006 to 2008–2009) [19,20,21,22,23], where ILI surveillance data and information about incidence by subtype are sparse. While we cannot analyze data from these seasons, we summarize the available evidence for differential VE for completeness. RCTs seldom measure vaccine efficacy against specific subtypes. Thus, to calculate average vaccine efficacies by subtype, we first substitute subtype-specific vaccine efficacy with overall vaccine efficacy (or efficacy against type A where applicable) when one type or subtype clearly dominates the cases in the study population. We calculate the average efficacy for each season, and then calculate overall average efficacies by subtype. On average from 2005–2006 to 2008–2009, RCTs show lower efficacy against H3N2 (54.7%, 95% CI: 41.8–64.3%) compared to H1N1 (73.6%, 95% CI: 60.3–77.5%), but unlike TND studies from later years, RCTs show comparable (if not slightly higher) efficacy against H3N2 compared to B (53.5%, 95% CI: 27.0–69.9%). These differences are not statistically significant. RCTs of the inactivated seasonal vaccine in children are even less common, take place before 2002, and do not measure efficacy against specific types/subtypes [1,78,79,80,81].

It is unclear whether differences in VE by subtype from the few RCTs from 2005–2006 to 2008–2009 are relevant to our analysis of more recent surveillance data (from 2009–2010 to 2016–2017). Canadian TND studies from a similar time period as RCTs show similar trends [32,33]: lower VE against H3N2 (57%, 95% CI: 42.6–67.7%) compared to H1N1 (80.1%, 95% CI: 64.9–88.6%), but higher VE against H3N2 compared to B (45.1%, 95% CI: 21.6–61.5%). However, the differences are again not statistically significant. For our analysis of the recent surveillance data, we base our expectations on the more recent TND studies.

## Appendix C. Derivation of Theoretical Subtype Ratios

The expected number of infections in an unvaccinated and non-immune population of size *N* with prevalence $\alpha =\frac{I}{I+S}$ is

(A15) $$\begin{array}{c} I=N\alpha . \end{array}$$

We include vaccination and define its impact *E* in terms of vaccine efficacy (1−riskratio) as opposed to vaccine effectiveness (1−oddsratio). When incidence is low, the two estimates approach each other. Vaccination status is indicated with a subscript v for vaccinated or a subscript u for unvaccinated:

(A16) $$\begin{array}{cc} E & =1-\frac{I_{v}/\left(I_{v}+S_{v}\right)}{I_{u}/\left(I_{u}+S_{u}\right)}. \end{array}$$

When some fraction *p* of the population is vaccinated, the expected number of infections is

(A17) $$\begin{array}{cc} I & =N\left(1-p\right)\alpha +Np\left[I_{v}/\left(I_{v}+S_{v}\right)\right]. \end{array}$$

Since the unvaccinated and vaccinated hosts belong to the same population, we can substitute α in the expression for *E* (Equation (A16)). Here, we also assume that there are no indirect effects of vaccination:

(A18) $$\begin{array}{cc} E & =1-\frac{I_{v}/\left(I_{v}+S_{v}\right)}{\alpha }, \end{array}$$

(A19) $$\begin{array}{cc} I_{v}/\left(I_{v}+S_{v}\right) & =\alpha (1-E). \end{array}$$

Substituting Equation (A19) into Equation (A17), we obtain

(A20) $$\begin{array}{cc} I & =N(1-p)\alpha +Np\alpha (1-E). \end{array}$$

For any two strains $s_{1}$ and $s_{2}$, the expected ratio of infections is

(A21) $$\begin{array}{cc} \frac{I_{s_{1}}}{I_{s_{2}}} & =\frac{N\left(1-p\right)\alpha_{s_{1}}+Np\alpha_{s_{1}}\left(1-E_{s_{1}}\right)}{N\left(1-p\right)\alpha_{s_{2}}+Np\alpha_{s_{2}}\left(1-E_{s_{2}}\right)} \end{array}$$

(A22) $$\begin{array}{c} =\frac{\alpha_{s_{1}}}{\alpha_{s_{2}}}\frac{1-p+p(1-E_{s_{1}})}{1-p+p(1-E_{s_{2}})} \end{array}$$

(A23) $$\begin{array}{c} =\frac{\alpha_{s_{1}}}{\alpha_{s_{2}}}\frac{1-pE_{s_{1}}}{1-pE_{s_{2}}}. \end{array}$$

Equation (A23) shows that the expected ratio of strain prevalences is proportional to the ratio of the effective fraction of the population that is unvaccinated against each subtype. The ratio of strain prevalences is expected to scale linearly with the ratio of the effective unvaccinated fractions. The expected change in the ratios of strain prevalences (for H3N2, H1N1, and B) with vaccine coverage based on VE measured in Canadian TND studies [27,28,29,30,31,32,33,34] is shown in Figure 1.

## Appendix D. Supplementary Tables and Figures

**Table A1:** Vaccine efficacy in adults measured in randomized control trials.

		Efficacy (CI)							Inclusion in Averages ${}^{a}$		
Ref.	Season	Overall	A	H3N2	H1N1	B	Type/Subtype Dominance	Location	H3N2	H1N1	B
[21]	2008	41.0 (21.1, 55.9)	–	–	–	–	∼50% B	Australia	(D)	(D)	(D)
[21]	2009	42.7 (26.3, 55.4)	–	–	–	–	H1N1 dominated	Australia	(N)	**I**	(N)
[82]	2004–2005	74 (37, 89)	–	–	–	–	50% H3N2 50% B	USA	(D)	(D)	(D)
[83]	2005–2006	22.3 (−49.1, 58.5)	25.1 (−260.9, 82.2)	–	–	21.5 (−65.9, 61.6)	Very few cases	Czechia	(F)	(F)	(F)
[84]	2005–2006	49.4 (12.7, 70.7)	–	–	–	–	Majority H3N2	USA	**I**	(D)	(D)
[82]	2005–2006	23 (−153, 73)	–	–	–	–	Majority H3N2	USA	(F)	(N)	(N)
[84]	2006–2007	49.2 (−0.04, 75.3)	–	–	–	–	Majority H3N2	USA	**I**	(D)	(D)
[20]	2006–2007	61.6 (46, 72.8)	–	–	–	–	>99% H3N2	Czechia, Finland	**I**	(N)	(N)
[85]	2007–2008	63.0 (46.7)	–	49.3 (−9)	81.5 (60.9)	53.2 (22.2)	–	USA, Finland, Poland	**I**	**I**	**I**
[22]	2007–2008	–	72 (49, 84)	–	–	40 (−189, 86)	90% H3N2	USA	**I**	(F)	(F)
[23]	2007–2008	–	49.0 (24.7, 65.9)	–	–	37.2 (−8.9, 64.5)	70% H3N2 among A	USA	**I**	(N)	**I**
[19]	2008–2009	71.5 (54.7, 82.1)	–	50.0 (−173, 90.8)	75.2 (55.4, 86.2)	60.1 (9.5, 82.4)	–	USA	(F)	**I**	**I**

${}^{a}$**I** Include in average (Table A2), (F) Too few cases, (D) Ambiguous type/subtype dominance, (N) Not reported and not estimable.

**Table A2:** Estimated seasonal vaccine efficacy in adults.

	Average Efficacy (Studies Used to Compute Average) ${}^{a}$		
Season	H3N2	H1N1	B
2005–2006	49.4 (12.7,70.7) [84]	–	–
2006–2007	55.9 (35.5,70.4) [20,84]	–	–
2007–2008	58.3 (40.9,66.9) [22,23,85]	81.5 (60.9) [85]	45.8 (21.2, 59.2) [23,85]
2008–2009	–	62.3 (48.1, 72.6) [19,21]	60.1 (9.5, 82.4) [19]
Average	54.7 (41.8, 64.3)	73.6 (60.3, 77.5)	53.5 (27.0, 69.9)

${}^{a}$ Seasonal averages are calculated using arithmetic means of log RRs (Equation (A7)) reported by studies listed in Table A1. When subtype or type-specific efficacy is not explicitly measured, the efficacy against the dominant type/subtype in the study population is estimated as equal to the overall efficacy.

**Table A3:** Evidence for potential vaccine-driven selection among influenza types and subtypes.

Seasons	Observation	References	Interpretation
2005–2006 to 2008–2009	Lower vaccine efficacy against H3N2 (54.7%, 95% CI: 41.8-64.3%) compared to H1N1 (73.6%, 95% CI: 60.3, 77.5%), though not a statistically significant difference. Similar efficacy against H3N2 relative to B (53.5%, 95% CI: 27.0, 69.9%) reported in randomized control trials (RCTs) in adults (Table A2).	[19,20,21,22,23]	Possible selection for H3N2 relative to H1N1 but not necessarily relative to B. More recent RCTs have not yet been reported.
2009–2010 to 2016–2017	Lower vaccine effectiveness against H3N2, compared to H1N1 and B based on test-negative design studies averaged over time. Differences are significant in Canada [27,28,29,30,31,32,33,34] and the United States [36,37,38,39,40,41,42,43], but not in Australia [24,25,26] for H3N2 vs. B, and not in Europe [35] for both H3N2 vs. B and H3N2 vs. H1N1 (Figure A1 and Figure A2).	[24,25,26,27,28,29,30,31,32,33,34,35,36,37,38,39,40,41,42,43]	Expect selection for H3N2 relative to relative to H1N1 and B.

**Table A4:** Evidence for potential vaccine-driven selection among influenza B lineages.

Observation	References	Interpretation
Vaccination with a Victoria strain (B/Brisbane/60/2008-like) induced antibodies responses against Victoria and Yamagata strains by HI, but vaccination with a Yamagata strain (B/Florida/4/2006-like) only elicits antibody responses against Yamagata (in mice and in children using the LAIV).	[66,67]	Possible selection for Victoria during Yamagata vaccine seasons. No vaccine-driven selection for either lineage during Victoria vaccine seasons.
The quadrivalent vaccine elicited significantly greater HI titers against both lineages than did the trivalent vaccine against the heterologous lineage.	[64,65]	Possible selection for the unmatched lineage.
TIV effectiveness against clinical influenza infection was comparable against both lineages in the 2011-2012, 2012-2013, and 2015-2016 seasons. Trivalent vaccines were effective against influenza B even in seasons dominated by a lineage that mismatches the vaccine.	[29,34,36,38,39]	Vaccine-driven selection for either lineage is not expected.

**Table A5:** Evidence for potential vaccine-driven selection among H3N2 strains.

Season	Observation	References	Interpretation
2016–2017	The egg-adapted vaccine strain lacks glycosylation site found in circulating 3c2.A viruses.	[86]	The vaccine is poorly immunogenic against circulating strains in general. Little vaccine-driven selection is expected.
2014–2015	Circulating 3c2.A viruses have a new glycosylation site and several other site B residues different from the vaccine strain. Clade-specific VEs differ: 3c2.A −13% (95% CI, −51% to 15%); 3c3.B 52% (95% CI, −17% to 80%)	[28,44,45]	3c2.A strains differ immunologically from the vaccine strain. Vaccine-driven selection for 3c2.A strains is expected.
2012–2013	In adults, the vaccine fails to induce responses to novel mutations on the vaccine strain’s HA.	[71]	The vaccine is poorly immunogenic against circulating strains in general. Little vaccine-driven selection is expected.
